# Indicators Measuring the Performance of Malaria Programs Supported by the Global Fund in Asia, Progress and the Way Forward

**DOI:** 10.1371/journal.pone.0028932

**Published:** 2011-12-19

**Authors:** Jinkou Zhao, Marcel Lama, Swarup Sarkar, Rifat Atun

**Affiliations:** 1 The Global Fund to Fight AIDS, Tuberculosis and Malaria, Vernier, Geneva, Switzerland; 2 Jiangsu Provincial Center for Disease Control and Prevention, Nanjing, China; 3 The United Nations Programme on HIV/AIDS, Geneva, Switzerland; 4 Imperial College London, London, United Kingdom; Johns Hopkins University, United States of America

## Abstract

**Introduction:**

In 2010, the Global Fund provided more than 75% of external international financing for malaria control. The Global Fund uses performance based funding in the grants it finances. This paper analyses the indicators used to measure the performance of Global Fund supported malaria grants in Asia.

**Methods:**

Indicators used in the performance frameworks for all Global Fund supported malaria grants in Asia were retrieved from grant database and grouped into impact, outcome, output and input categories and categorized by service delivery areas. Indicators of each group were compared over rounds. Indicators used in performance frameworks were compared with internationally adopted indicators included in the Monitoring and Evaluation Toolkit developed by the Global Fund and international technical agencies.

**Results:**

Between 2002 and 2010, 1,434 indicators were included in the performance frameworks of the 48 malaria grants awarded in Asia, including 229 impact and 227 outcome indicators, 437 output and 541 input indicators, with an average of 29.9 indicators per grant. The proportion of impact and outcome indicators increased over rounds, with that of input indicators declining from 44.1% in Round 1 to 22.7% in Round 9.

**Conclusions:**

Input indicators, which have predominated the performance frameworks of the Global Fund supported malaria programs in Asia have declined between Rounds 1 and 9. However, increased alignment with internationally adopted indicators included in the Monitoring and Evaluation Toolkit is needed to improve the validity of reported results.

## Introduction

International financing for malaria control increased by 166%, from US Dollars ($) 0·73 billion in 2007 to $1·94 billion by 2009 [1]. The Global Fund to fight AIDS, Tuberculosis and Malaria (the Global Fund) accounted for 75% of international financing [2], with $5.3 billion committed for malaria programs in 83 countries, including $0.95 billion in Asia covering 32 countries in East Asia and the Pacific and South and West Asia [Box S1].

Malaria epidemiology is highly heterogeneous in Asia [3]: endemic in the south and west Asian and the Pacific countries, highly focal in the countries and areas of the Greater Mekong sub-region, such as Cambodia, Yunnan province of China, the Lao People's Democratic Republic and Viet Nam, restricted to particular geographical locations in Malaysia, the Philippines and the Republic of Korea, and no indigenous transmission in the Maldives since 1984. Most countries have both *Plasmodium* (*P.*) *falciparum* and *P. vivax*. Transmission in Afghanistan, North and South Korea, Sri Lanka and central areas of China is primarily due to *P. vivax* [3].

The Global Fund uses performance based funding when investing in AIDS, tuberculosis, malaria and health systems strengthening. Grants are implemented in two phases: the phase one for two years and the phase two for three years. Performance in the phase one determines funding for the phase two. Grant performance is measured quarterly or six monthly, when disbursements are made adjusted by performance. The principal recipient (PR) and the Global Fund jointly develop a performance framework [4]: a legally binding agreement signed by each, to monitor grant performance. The performance framework comprises indicators, targets, data sources and reporting requirements reflecting goals and objectives of the grant, local epidemiology, and strength of the local reporting systems. The PR and the Global Fund can agree to revise the performance framework following the first phase of program implementation reflecting performance, evolving epidemiology and contextual factors.

A performance framework typically includes 2 to 5 impact and outcome indicators to measure achievement of program goals and objectives, and up to 15 ‘programmatic’ input and output indicators to measure progress with major activities, which are grouped as service delivery areas (SDAs). Different categories of indicators are shown in Table 1. Each indicator has a time-bound target, with targets for impact and outcome indicators set for 1 or 2-year periods to assess performance at end of the phase one [5], and programmatic indicators set for 3 to 6 months and used to assess implementation performance for the period in question and to determine disbursement for the next period. To guide PRs in indicator selection and to ensure consistency of indicator wording and comparability of results across grants the Global Fund developed a Monitoring and Evaluation Toolkit (M&E Toolkit) with its technical and financing partners in 2004, with revisions in 2006, 2009 and 2011 emphasizing internationally adopted indicators used to measure outcome and impact [6].

**Table 1:** Indicator categories in the performance framework, with examples.

Indicator categories	Measurement areas	Frequency of measurements	Data source	Examples
Impact	Disease mortality or morbidity	Every 3–5 years	Population-based surveys or routine health information system; such as demographic health survey or vital registration	All-cause mortality rate among children younger than 5 years of age Slide or rapid diagnostic testing positivity rate: people found positive in slide or rapid diagnostic testing among all slides or rapid diagnostic tests taken
Outcome	Behavioral change	Every 3–5 years	Population-based surveys, such as demographic health survey	Percentage of children younger than 5 years of age who slept under an insecticide-treated net the previous night Percentage of children younger than 5 years of age (or other target age groups) with fever in the last 2 weeks who received any antimalarial treatment
Output	Target population reached by key interventions	Quarterly, semi-annually or annually	Programmatic data, facility records	Number of insecticide-treated nets distributed to people Number of confirmed malaria cases treated according to national policy
Input	Finance or resource investment	Quarterly, semi-annually or annually	Programmatic data, facility records	Number of people attended advocacy meetings Number of districts with increased financial contribution for malaria intervention

Grant performance is rated according to achievements towards targets set in the performance framework. Performance rating informs disbursement decisions and funding awarded for the second phase of the grant [7], [8]. We analyze malaria programs supported by the Global Fund in in Asia, to explore for the first time the indicators used in performance frameworks to measure grant performance and the alignment of these indicators with internationally adopted indicators defined in the M&E Toolkit.

## Methods

### Data sources

We used the Global Fund grants database to identify and tabulate indicators included in performance frameworks (both Phase one and two) of all Round 1–9 malaria grants supported by the Global Fund in Asia over the period 2002 and 2010, and financing for these grants.

### Data analysis

We grouped indicators into impact, outcome, output and input categories, then sub-grouped all but impact indicators according to SDAs (see Table S1 for a list of SDAs). We computed the number and proportion of indicators in each category and SDA, and the cumulative funding allocated to each SDA, comparing them over Rounds 1–9.The analysis is limited to the indicators included in the performance frameworks, does not include the actual reported results against the targets for the indicator.

The indicators included in the performance frameworks were assessed against the definition and wording of relevant indicators in the M&E Toolkit 2009 version and categorized as ‘aligned’, ‘partially aligned’ and ‘not aligned’ – aligned if an indicator in the performance framework matched that in the M&E Toolkit, partially aligned if key elements of an indicator were expressed using different wording to that in the Toolkit, and non aligned if the indicator used in the performance framework was totally different or not included in the M&E Toolkit. Alignment of indicators with M&E Toolkit was compared over rounds by SDAs and by indicator category using SPSS 13.0 (SPSS Inc., Chicago, IL). Examples of indicators with relevant categorization are shown in Box S2.

## Results

Between 2002 and 2010, from Round 1 to Round 9, the Global Fund approved in Asia 48 malaria grants with a budget of $950 million. There were 1,434 indicators included in the performance frameworks of these 48 grants: comprising 229 impact and 227 outcome indicators, 437 output and 541 input indicators (Table S1).

While the number and proportion of indicators used in the SDAs relating to prevention, treatment and health system strengthening (HSS) this difference had no clear trend (P = 0.007). The top five SDAs accounted for 80% of the indicators: 23.6% (284/1205) for activities relating to insecticide treated nets (ITNs), 17.3% for facility and home treatment, 16.3% for training, 11.7% for diagnosis, 10.9% for behavioral communication change. Only two of the 1,205 indicators were included in the performance frameworks for activities related to indoor residual spraying, an intervention critically important in low malaria transmission areas.

**Figure 1:**
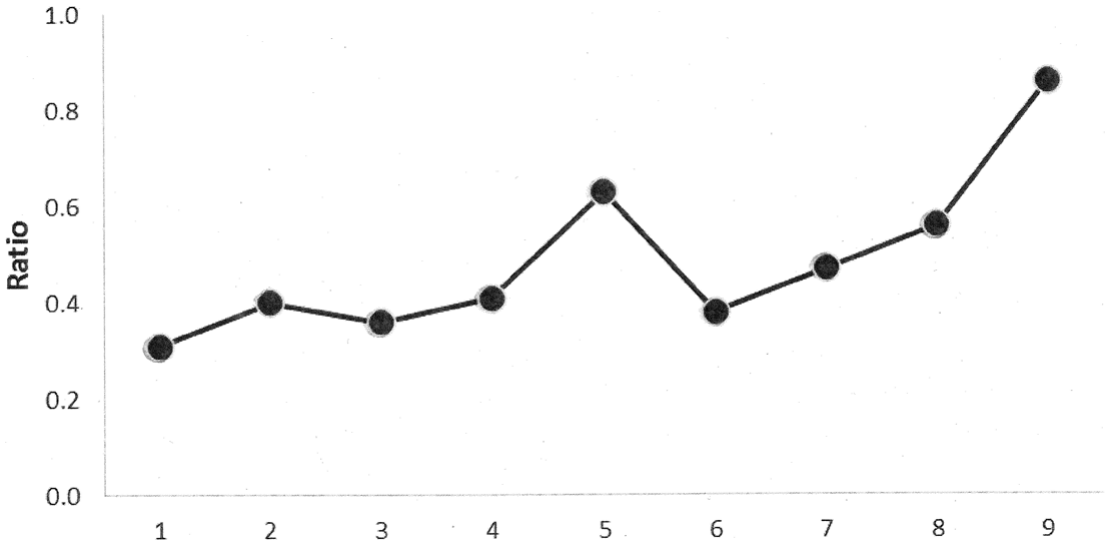
Ratio of the number of impact and outcome indicators to the number of output and input indicators from Round 1 to 9.

The number of impact and outcome indicators increased over Rounds 1 to 9 (P = 0.007), with a rise in the ratio of these over output and input indicators (P = 0.002) (Figure 1). However, while the input indicators declined in proportion to the total number of indicators, by Round 9 these still accounted for 38% (range of 23% to 44% across rounds) of total.

**Figure 2:**
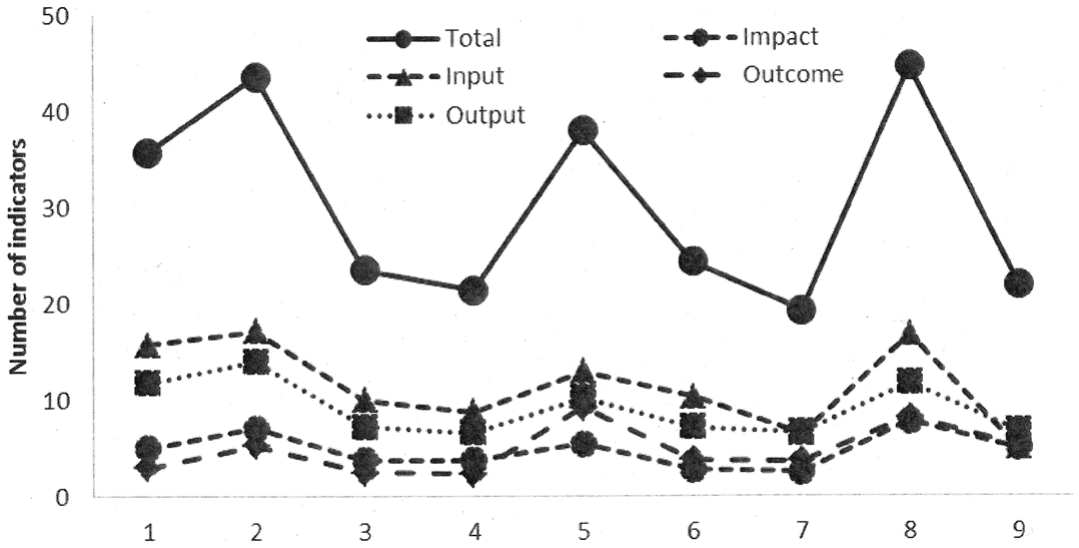
Average number of indicators of different categories from Rounds 1 to 9.

The average number of indicators per grant, which remained constant over rounds, was 29.9 (range of 19.3 to 44.6): 4.8 (range 2.8 to 7.8) for impact, 4.7 (range 2.5 to 9.3) for outcome, 9.1 (range of 6.6 to 14.0) for output and 11.3 (range 5.0 to 17.1) for input indicators. (Figure 2)

Input indicators accounted for 37.7% over the rounds, decreasing in t proportion from 44.1% of total in Round 1 to 22.7% in Round 9 (Figure 2, P = 0.000).

Indicators related to training accounted for 16.3% of the total (196/1,205), declining from 26.4% in Round 1 to 9.3% in Round 5 (Table S1) increasing thereafter, but without an obvious trend.

Of the 1,434 indicators used, 43.2% was aligned, 28.0% partially aligned and 28.8% not aligned with the indicators used in the M&E Toolkit. The proportion of aligned indicators increased over the rounds, with a decline in the proportions of partially aligned and not aligned indicators (P = 0.000). While the indicators for prevention (P = 0.025) and treatment (P = 0.018) SDAs were increasingly aligned over Rounds 1–9, those for HSS did not change (P = 0.380) with 41.5% of these indictors not aligned with the M&E Toolkit.

Over the rounds, the number of indicators relating to ITNs (P = 0.024) and facility and home treatment (P = 0.003) were increasingly aligned, unlike those relating to coordination and supportive environment where 94% of indicators remained ‘not aligned (P = 0.001) (Table 2).

**Table 2:** Alignment of indicators in the performance framework with M&E Toolkit, by service delivery areas (SDA), over the rounds.

SDA	Alignment	Rounds									Total	P value
		1	2	3	4	5	6	7	8	9		
Impact	Aligned	5	16	2	9	9	3	10	27	11	92	0.000
	Partially aligned	11	19	7	9	12	11	10	10	8	97	
	Not aligned	6	15	6	1	1	3	0	2	6	40	
	Sub total	22	50	15	19	22	17	20	39	25	229	
Health system strengthening	Aligned	23	32	13	11	11	16	20	24	7	157	0.380
	Partially aligned	9	7	6	5	3	6	4	6	4	50	
	Not aligned	3	15	37	6	9	16	21	11	27	147	
	Sub total	47	76	25	25	30	43	35	57	16	354	
Prevention	Aligned	15	53	17	24	47	22	41	53	25	297	0.025
	Partially aligned	11	26	8	13	7	8	7	10	7	97	
	Not aligned	11	23	8	9	9	5	15	11	3	94	
	Sub total	37	102	33	46	63	35	63	74	35	488	
Treatment	Aligned	2	20	3	0	7	10	14	9	9	74	0.018
	Partially aligned	2	24	28	10	9	15	19	17	21	157	
	Not aligned	11	29	8	8	15	22	5	23	11	132	
	Sub total	37	77	21	17	37	51	36	53	34	363	
Total	Aligned	45	121	35	44	74	51	85	113	52	620	0.000
	Partially aligned	55	80	31	36	37	44	38	47	33	401	
	Not aligned	43	104	28	27	41	51	31	63	25	413	
	Sub total	143	305	94	107	152	146	154	223	110	1,434	

Overall alignment of input (P = 0.373) and output indicators (P = 0.108) did not improve over the rounds, with only 31.1% and 41.6% respectively aligned with M&E Toolkit. In contrast the alignment of impact (P = 0.000) and outcome (P = 0.052) indicators improved over time (Table 3).

**Table 3:** Compliance of indicators in the performance framework with M&E Toolkit, by indicator categories, over the rounds.

Indicator categories	Alignment	Rounds									Total	P value
		1	2	3	4	5	6	7	8	9		
Impact	Aligned	5	16	2	9	9	3	10	27	11	92	0.000
	Partially aligned	11	19	7	9	12	11	10	10	8	97	
	Not aligned	6	15	6	1	1	3	0	2	6	40	
	Sub total	22	50	15	19	22	17	20	39	25	229	
Outcome	Aligned	5	27	9	9	29	17	24	37	21	178	0.052
	Partially aligned	4	5	1	3	4	1	1	4	2	25	
	Not aligned	3	5	0	0	4	5	4	0	3	24	
	Sub total	12	37	10	12	37	23	29	41	26	227	
Output	Aligned	11	43	11	13	25	15	29	24	11	182	0.108
	Partially aligned	26	36	11	15	11	21	16	20	17	173	
	Not aligned	9	19	7	5	5	8	8	15	6	82	
	Sub total	46	98	29	33	41	44	53	59	34	437	
Input	Aligned	24	35	13	13	11	16	22	25	9	168	0.373
	Partially aligned	14	20	12	9	10	11	11	13	6	106	
	Not aligned	25	65	15	21	31	35	19	46	10	267	
	Sub total	63	120	40	43	52	62	52	84	25	541	
Total	Aligned	45	121	35	44	74	51	85	113	52	620	0.000
	Partially aligned	55	80	31	36	37	44	38	47	33	401	
	Not aligned	43	104	28	27	41	51	31	63	25	413	
	Sub total	143	305	94	107	152	146	154	223	110	1,434	

For several SDAs, there was a clear asymmetry between the funding allocated and the number of indicators used to assess the performance (Table 4). For example, while 0.3% of the total budget was allocated to training over the period 2002–2010, the number of indicators for training accounted for 16.3% of the total (196/1,434). For diagnosis SDA, which accounted for 3.9% of the total budget the number of indicators were 12.7% of the total, similar to treatment SDA, which accounted for 17.2% of the total budget ($163 million) with 30.1% of the total indicators.

**Table 4:** Proportional relationship between budget and indicators by service delivery areas.

Service delivery areas	Budget	Number of indicators
Prevention	466,806,232	488
Behavioral change and communication, other prevention	132,999,464	129
Insecticide treated nets	323,766,410	282
Indoor residual spray	9,015,718	33
Prevention in pregnancy	1,024,640	44
Treatment	163,214,517	363
Diagnosis	37,224,344	144
Facility and home treatment	114,832,293	209
Drug resistance	11,157,720	10
Health system strengthening	317,269,653	354
Monitoring and evaluation	14,698,430	89
Coordination and supportive environment	300,202,440	69
Training	2,368,782	196
Total	947,383,974	1,205

## Discussion

We present the first analysis of indicators used in performance frameworks of Global Fund financed malaria programs and their alignment with internationally adopted indicators. Achievements against the targets set using these indicators determine the amount of funding received by grants. The indicators used in performance frameworks and the targets for these indicators form the basis for performance-based funding, and financing of grants. Hence, appropriate selection and definition of indicators is critical to ensure performance is appropriately measured and well performing grants duly rewarded. Inappropriate indicators may distort the performance rating and therefore grant funding.

Since 2009, renewed emphasis by the Global Fund on measurement of outcomes and impact of its investments has strengthened performance measurement of malaria grants in Asia, with increased use of impact and outcome indicators in grant performance frameworks. However, a large proportion of indicators in grant performance frameworks are still input indicators, especially those for training activities, reflecting poor attention to indicator selection in the period preceding 2009, giving undue emphasis in performance of SDAs driven by inputs rather than outcomes and thereby skewing financing towards grants that have achieved improved inputs but not necessarily outcomes or impact [8]. Hence, when negotiating performance frameworks with countries, the Global Fund will continue to reduce the number of indicators per grant and focus them on output, outcomes and impact. Additional analysis with actual program results is needed to quantify how the composition of indicators within the performance framework affects grant performance rating.

Although in Asia the malaria epidemic is heterogeneous, in the majority of Asian countries malaria remains localized. While massive ITN distribution would be an effective strategy in high malaria transmission areas, IRS would be especially effective in low malaria transmission areas [9], [10]. However, in Asia in the malaria grants supported by the Global Fund indicators for ITN are the most frequently monitored indicators, rather than those for IRS at odds with the prevailing epidemiology [11].

Alignment of indicators with the Global Fund M&E Toolkit increased in each successive round, especially with impact and outcome indicators. Consistency of indicator definition and wording across different grants is critical for the data aggregation at regional or global level and comparison across the regions or different epidemic situations. Malaria indicators in the current M&E Toolkit are more relevant to high transmission areas such as Africa where large majority of Global Fund investments are made, with fewer indicators relevant to low transmission areas, including those at pre-elimination and elimination stages which would be more relevant for Asia. The revised M&E Toolkit due for release in 2011 will expand indicators suited to low transmission countries and those in pre-elimination stage, which will provide further flexibility for PRs to select indicators relevant to the epidemic stage in the country.

Different descriptions of the same indicator result in duplication and create difficulty in comparing results. Standardization of each indicator in the new M&E Toolkit will improve the validity of performance framework as well as the consistency and comparability of results across rounds and regions/countries.

In conclusion, an improvement has been observed in the Global Fund performance frameworks for malaria grants in Asia from round 1 to round 9, as evident by decreased proportion of input indicators and increased proportion of outcome and impact indicators. Efforts shall still be made to select indicators, appropriate in the total number per grant and allocation of categories, to ensure the performance framework is measuring in a standardized way what it supposes to measure, and therefore improve the value for money of the Global Fund investments in malaria programs in Asia and in the world.

## Supporting Information

Table S1 **Indicators in performance framework of malaria grants in Asia from Round 1 through Round 9, by service delivery areas (SDA) over the rounds.**
(DOC)

Box S1 **List of countries in the Asia region.**
(DOC)

Box S2 **Alignment of different indicators in performance framework with M&E Toolkit.**
(DOC)
