# Supplementary material for: Indicators Measuring the Performance of Malaria Programs Supported by the Global Fund in Asia, Progress and the Way Forward
Source: PLoS One. 2011 Dec 19;6(12):e28932. doi: 10.1371/journal.pone.0028932 (PMC3242766; doi:10.1371/journal.pone.0028932)
Supplement: Table S1 — Indicators in performance framework of malaria grants in Asia from Round 1 through Round 9, by service delivery areas (SDA) over the rounds. (DOC) [file pone.0028932.s001.doc]

Table S1. Indicators in performance framework of malaria grants in Asia from Round 1 through Round 9, by service delivery areas (SDA) over the rounds

| Indicators categories | SDA | Round | | | | | | | | | Total |
| --- | --- | --- | --- | --- | --- | --- | --- | --- | --- | --- | --- |
|  |  | 1 | 2 | 3 | 4 | 5 | 6 | 7 | 8 | 9 |  |
| Number of grants | | 4 | 7 | 4 | 5 | 4 | 6 | 8 | 5 | 5 | 48 |
| Number of indicators per grant | | 35.8 | 43.6 | 23.5 | 21.4 | 38.0 | 24.3 | 19.3 | 44.6 | 22.0 | 29.9 |
| **Impact indicators** | | 22 | 50 | 15 | 19 | 22 | 17 | 20 | 39 | 25 | 229 |
| **Outcome indicators** | *Prevention* | 11 | 33 | 10 | 12 | 30 | 20 | 25 | 37 | 21 | 199 |
|  | Behavioral change and communication | 1 | 0 | 0 | 0 | 0 | 0 | 1 | 0 | 0 | 2 |
|  | Insecticide treated nets | 9 | 25 | 7 | 12 | 18 | 16 | 18 | 24 | 18 | 147 |
|  | Indoor residual spray | 1 | 1 | 1 | 0 | 2 | 1 | 4 | 3 | 1 | 14 |
|  | Prevention in pregnancy | 0 | 7 | 2 | 0 | 10 | 3 | 2 | 10 | 2 | 36 |
|  | *Treatment* | 1 | 4 | 0 | 0 | 7 | 3 | 4 | 4 | 5 | 28 |
|  | Diagnosis | 0 | 0 | 0 | 0 | 0 | 0 | 0 | 0 | 3 | 3 |
|  | Facility and home treatment | 1 | 4 | 0 | 0 | 7 | 3 | 4 | 4 | 2 | 25 |
|  | Subtotal | 12 | 37 | 10 | 12 | 37 | 23 | 29 | 41 | 26 | 227 |
| **Output indicators** | *Prevention* | 18 | 42 | 17 | 21 | 23 | 13 | 26 | 27 | 12 | 199 |
|  | Behavioral change and communication | 4 | 9 | 3 | 3 | 7 | 4 | 5 | 11 | 5 | 51 |
|  | Insecticide treated nets | 10 | 27 | 9 | 16 | 14 | 9 | 17 | 15 | 6 | 123 |
|  | Indoor residual spray | 2 | 4 | 3 | 2 | 2 | 0 | 4 | 0 | 1 | 18 |
|  | Prevention in pregnancy | 2 | 2 | 2 | 0 | 0 | 0 | 0 | 1 | 0 | 7 |
|  | *Treatment* | 26 | 56 | 12 | 11 | 18 | 30 | 25 | 32 | 22 | 232 |
|  | Diagnosis | 10 | 14 | 4 | 3 | 4 | 13 | 10 | 15 | 8 | 81 |
|  | Facility and home treatment | 16 | 42 | 8 | 8 | 14 | 17 | 15 | 17 | 14 | 151 |
|  | *Health system strengthening* | 3 | 0 | 0 | 1 | 0 | 0 | 2 | 0 | 0 | 6 |
|  | Monitoring and evaluation | 1 | 0 | 0 | 0 | 0 | 0 | 0 | 0 | 0 | 1 |
|  | Coordination and supportive environment | 2 | 0 | 0 | 1 | 0 | 0 | 2 | 0 | 0 | 5 |
|  | Subtotal | 47 | 98 | 29 | 33 | 41 | 43 | 53 | 59 | 34 | 437 |
| **Input indicators** | *Prevention* | 10 | 28 | 6 | 13 | 11 | 2 | 12 | 10 | 2 | 94 |
|  | Behavioral change and communication | 8 | 25 | 4 | 10 | 9 | 1 | 11 | 9 | 1 | 78 |
|  | Insecticide treated nets | 2 | 3 | 2 | 3 | 1 | 1 | 1 | 0 | 1 | 14 |
|  | Indoor residual spray | 0 | 0 | 0 | 0 | 0 | 0 | 0 | 1 | 0 | 1 |
|  | Prevention in pregnancy | 0 | 0 | 0 | 0 | 1 | 0 | 0 | 0 | 0 | 1 |
|  | *Treatment* | 9 | 18 | 9 | 6 | 11 | 17 | 7 | 17 | 7 | 100 |
|  | Diagnosis | 5 | 10 | 8 | 3 | 8 | 5 | 3 | 12 | 3 | 57 |
|  | Facility and home treatment | 4 | 5 | 1 | 2 | 0 | 12 | 1 | 5 | 4 | 33 |
|  | Drug resistance | 0 | 3 | 0 | 1 | 3 | 0 | 3 | 0 | 0 | 10 |
|  | *Health system strengthening* | 47 | 76 | 25 | 25 | 30 | 43 | 33 | 57 | 16 | 354 |
|  | Monitoring and evaluation | 6 | 20 | 5 | 5 | 7 | 11 | 7 | 22 | 6 | 89 |
|  | Coordination and supportive environment | 9 | 18 | 1 | 3 | 11 | 12 | 2 | 9 | 2 | 69 |
|  | Training | 32 | 38 | 19 | 17 | 12 | 20 | 24 | 26 | 8 | 196 |
|  | Subtotal | 63 | 120 | 40 | 44 | 52 | 62 | 52 | 84 | 25 | 541 |
| Subtotal by SDAs | Prevention | 37 | 102 | 33 | 46 | 63 | 35 | 63 | 74 | 35 | 488 |
|  | Treatment | 37 | 77 | 21 | 17 | 36 | 52 | 36 | 53 | 34 | 363 |
|  | Health system strengthening | 47 | 76 | 25 | 25 | 30 | 43 | 35 | 57 | 16 | 354 |
| Total | | 143 | 305 | 94 | 107 | 152 | 146 | 154 | 223 | 110 | 1434 |
