# Supplementary material for: Indicators Measuring the Performance of Malaria Programs Supported by the Global Fund in Asia, Progress and the Way Forward
Source: PLoS One. 2011 Dec 19;6(12):e28932. doi: 10.1371/journal.pone.0028932 (PMC3242766; doi:10.1371/journal.pone.0028932)
Supplement: Box S1 — List of countries in the Asia region. (DOC) [file pone.0028932.s002.doc]

**Box S1. List of countries in the Asia region**

Asia region defined by the Global Fund includes Afghanistan, Bangladesh, Bhutan, Cambodia, China, India, Indonesia, Iran, North Korea, Laos, Myanmar, Malaysia, Nepal, Pakistan, Papua New Guinea, Philippines, Sri Lanka, Thailand, Timor-Leste, Viet Nam and Pacific island countries Cook Islands, Fiji, Micronesia (Federated States), Kiribati, Niue, Marshall Islands, Palau, Samoa, Solomon Islands, Tonga, Tuvalu and Vanuatu.
