# Supplementary material for: Indicators Measuring the Performance of Malaria Programs Supported by the Global Fund in Asia, Progress and the Way Forward
Source: PLoS One. 2011 Dec 19;6(12):e28932. doi: 10.1371/journal.pone.0028932 (PMC3242766; doi:10.1371/journal.pone.0028932)
Supplement: Box S2 — Alignment of different indicators in performance framework with M&E Toolkit. (DOC) [file pone.0028932.s003.doc]

**Box S2. Alignment of different indicators in performance framework with M&E Toolkit**

To guide the development of Performance Frameworks in the grants it finances, the Global Fund developed the Monitoring and Evaluation (M&E) Toolkit, which recommends the impact, outcome and output indicators. To assess how M&E Toolkit plays the role as the guideline, indicators in the present analysis are compared with those recommended in the M&E Toolkit. Here are the examples if the indicators in the Performance Framework are aligned with those recommended in the M&E Toolkit.

Impact indicators

M&E Toolkit: Deaths due to malaria (confirmed malaria diagnosis) (percentage)

Aligned：Number and percentage of reported deaths due to malaria at health facilities

Partially aligned：Malaria death per 1000 population

Not aligned：% of confirmed malaria deaths among severe malaria cases

Outcome indicators

M&E Toolkit: Households with at least one insecticide-treated net (percentage)

Aligned: Number and percentage of households in 5 target provinces owning at least one ITN.

Partially aligned: % of general population living in high malaria endemic areas that have sufficient treated bed nets the previous night.

Not aligned: Number of persons protected by Long Lasting Insecticide Treated Nets

Output indicators

M&E Toolkit: Insecticide-treated nets (ITN) or re-treatment kits distributed to people (number)

Aligned: Number of LLINs (long lasting insecticide treated nets) distributed to the community

Partially aligned: Number of LLINs sold to beneficiaries through social marketing

Not aligned: Number of new outlets selling re-treatment tablets

Input indicators

M&E Toolkit: Health workers (including volunteers) trained for providing malaria services (number)

Aligned: Number of service providers trained in treatment of nets

Partially aligned: Number of service providers trained

Not aligned: Number of staff who received technical training overseas
